# Psychometric Properties of the Spanish Version of the VIA-72 Strengths Inventory

**DOI:** 10.3390/ejihpe15070129

**Published:** 2025-07-10

**Authors:** Francisco Varela-Figueroa, María García-Jiménez, Rosario Antequera-Jurado, Francisco Javier Cano-García

**Affiliations:** 1 Departamento de Personalidad, Evaluación y Tratamiento Psicológicos, Universidad de Sevilla, c/Camilo José Cela, 41018 Sevilla, Spain; fravarfig@alum.us.es (F.V.-F.); raj@us.es (R.A.-J.); 2 Departamento de Psicología Experimental, Universidad de Sevilla, c/Camilo José Cela, 41018 Sevilla, Spain; mgarciaj@us.es

**Keywords:** character strengths, VIA-72, psychometric validation, factor structure, personality traits, Spanish adaptation, positive psychology, confirmatory factor analysis

## Abstract

The Values in Action Inventory (VIA) is one of the most widely used measures for assessing character strengths. While the original version includes 240 items, shorter versions such as the VIA-72 have been developed to enhance its applicability. Psychometric studies of the VIA-72 in Spanish are still limited. This study examined the factorial structure, reliability, and convergent validity of the Spanish VIA-72 in a sample of 470 adults. Three alternative models—comprising three, five, and six factors—were tested using confirmatory factor analysis. All models showed acceptable fit, but the three-factor solution—Caring, Self-Control, and Inquisitiveness—showed the best performance in terms of parsimony, fit indices, and conceptual clarity. Internal consistency for the three-factor model was high across dimensions and comparable to previous studies. Convergent validity was supported through meaningful correlations with personality traits, particularly with conscientiousness. The factorial structure largely replicated findings obtained with both VIA-72 and VIA-240. These results support the Spanish VIA-72 as a reliable and valid instrument for assessing character strengths, offering a concise, theory-based alternative for Spanish-speaking populations.

## 1. Introduction

The emergence of positive psychology has marked a paradigm shift in the field of psychology, emphasizing the scientific study of human strengths, virtues, and optimal functioning (Seligman & Csikszentmihalyi, 2000). Within this framework, the exploration of character strengths and virtues has become a central area of research, aiming to understand and foster the traits that contribute to individual and collective well-being. This focus has roots in philosophical traditions but has been systematically studied through empirical methods, particularly since the seminal work of Peterson and Seligman (2004).

Peterson and Seligman’s Values in Action (VIA) classification provides a comprehensive framework for conceptualizing and measuring virtues and character strengths. The VIA model identifies six core virtues and twenty-four character strengths. It offers a universal taxonomy that bridges ancient philosophical traditions and modern psychological research. The core virtues are conceptual meta-categories, derived from cross-cultural and historical analyses, but the character strengths are measurable traits (Peterson & Seligman, 2004). The main difference between personality traits and character strengths is that the latter are intrinsically positive, represent moral excellence, and contribute to well-being, for which meta-analytic evidence is already available (Heintz et al., 2019; Linley, 2013). Studies have shown both convergence and distinctiveness between traits and strengths, highlighting the VIA’s unique contribution to understanding human behavior (McGrath, 2015). Thus, many studies on character strengths have used personality traits to validate their constructs and instruments. In general, with some exceptions, character strengths correlate negatively with neuroticism and positively with the other personality traits like extraversion, agreeableness, openness to experience, conscientiousness, or honesty (Anjum & Amjad, 2021; Littman-Ovadia & Lavy, 2012; McGrath et al., 2018).

The VIA Inventory of Strengths (VIA-IS) was developed to operationalize this framework, offering a reliable self-report tool for assessing the twenty-four character strengths. The VIA-IS uses 240 5-point Likert-style items (10 per strength scale) to measure the degree to which respondents endorse items reflecting their strengths. Scores are formed by averaging responses within scales, with higher numbers reflecting more strength.

Although there was originally no intention to empirically derive strengths from virtues, some studies have confirmed the six-factor structure initially proposed (Ruch et al., 2021; Ruch & Proyer, 2015). However, this has not always been the case. McGrath has conducted important studies on the VIA-IS latent structure (McGrath, 2014, 2015, 2023; McGrath et al., 2018; McGrath & Brown, 2020; McGrath & Wallace, 2021). First, he carried out impressive item-level and scale-level analyses from a large sample (N = 458,998) of U.S. adults (McGrath, 2014). The item-level analyses suggested an alternate set of 24 reliable scales, although only 20 of which overlapped substantially with existing VIA-IS scales. A second-order analysis suggested five factors, versus the original six virtues proposed in the development of the VIA-IS; seven interpersonal strengths (e.g., fairness, forgiveness); five emotional strengths (e.g., humor, social intelligence); four intellectual strengths (e.g., love of learning, curiosity); four restraint strengths (e.g., judgment, perspective); and four future-orientation strengths (e.g., positivity, future-mindedness). Second, a three-factor structure has been found from larger samples (McGrath, 2015; McGrath et al., 2018): Caring, most strongly associated with emotional and interpersonal strengths (e.g., gratitude, teamwork); Self-Control, characterized by strengths that have to do with one’s ability to function effectively in the world (e.g., prudence, perseverance); and Inquisitiveness, which was most highly related to those scales reflecting intellectual endeavors (e.g., creativity, curiosity).

Since its creation, the VIA-IS has gone through several iterations to improve its psychometric properties and applicability (McGrath, 2023). Although other measures like the Character Strengths Rating Form (CSRF) (Ruch et al., 2014), the Character Strengths Scale (CSS) (Noronha et al., 2015), the Brief Strengths Scale (BSS-12) (Ho et al., 2016), or the Adapted Inventory of Virtues and Strengths (AIVS) (Kim et al., 2022) have been developed, the VIA-IS has become the most widely used measure (Aluri & Kelly-Hedrick, 2023). It has been adapted cross-culturally to various countries and languages, such as Japan (Otake et al., 2005), the United Kingdom (Linley et al., 2007), Germany (Ruch et al., 2010), India (Singh & Choubisa, 2010), China (Duan et al., 2011), Israel (Littman-Ovadia & Lavy, 2012), Pakistan (Anjum & Amjad, 2020), and Spain (Azañedo et al., 2014). As for the latter, it was based on the responses of 1060 university students, finding five factors: interpersonal strengths, emotional strengths, theological strengths, strengths of restraint, and intellectual strengths. Although the reliability of the five factors was not reported, the reliability of the twenty-four individual strengths was acceptable to high (α = 0.73–0.88). Convergent validity was explored by correlations with life satisfaction, positive affect, and negative affect.

The original VIA-IS could be too long for practical purposes in many contexts, so the 240-item version was followed by shorter adaptations such as the VIA-120 (5 items per strength scale) and VIA-72 (3 items per strength scale), each designed to balance comprehensiveness with practical utility (McGrath & Wallace, 2021). The most recent VIA-IS-R/M/P, with 192 items (8 per strength scale), incorporates advances in measurement theory, demonstrating improved reliability and validity across diverse populations (McGrath, 2023). These advances include the use of both positively and negatively keyed items, item selection based on item response theory, prototypicality ratings, and improved unidimensionality and measurement invariance. The only adaptation in Spain of an abbreviated version (VIA-IS-120) was carried out by Azañedo et al. (2017) based on the responses of 2143 university students, finding five factors: interpersonal strengths, emotional strengths, theological strengths, strengths of restraint, and intellectual strengths. Although the reliability of the five factors was not reported, the reliability of the twenty-four individual strengths was acceptable to high (α = 0.72–0.88). Convergent validity was explored by correlations with life satisfaction, positive affect, and negative affect. However, as was the case with the study conducted by the same group to validate the VIA-IS version with the Spanish population (Azañedo et al., 2014), the use of a sample of university students somewhat hindered the generalization of the data to the Spanish population level.

The VIA-72 presents a reasonable balance between length and psychometric strength. It was derived from the original VIA-IS by extracting the three most internally consistent items from each scale. The internal consistency reliability is 0.75 on average, and initial validity coefficients range from 0.60 to 0.87 (https://www.viacharacter.org/researchers/assessments/via-72, accessed on 7 July 2025). Adaptations of the VIA-72 into other languages have started to expand its global applicability.

Very few studies have addressed the validity and reliability of the VIA-72. Moreira et al. (2022) confirmed the three-factor structure (Self-Control, Caring, and Inquisitiveness) in a sample of 509 Portuguese university students. The intercorrelations between the three factors were high (0.63–0.65). Convergent validity was explored by correlations with temperament and character traits derived from Cloninger’s theory.

Rodríguez Rueda (2024) reviewed the psychometric properties of the VIA-72 in a sample of 521 Colombians from the general population. The confirmatory factor analysis showed an adequate fit to the original six virtues model. The results showed adequate and high internal consistency according to Cronbach’s alpha (0.76–0.83) and McDonald’s omega (0.71–0.86). Convergent validity was assessed through positive correlations with problem-solving coping, mindfulness, resilience, and life satisfaction.

Finally, only one study has found a four-factor structure in the VIA-72 (Bhattarai et al., 2021): intellectual and emotional strengths, temperance, transcendence, and interpersonal strengths. However, the last factor was included for statistical reasons and, in our opinion, presents interpretation problems; for example, the strengths load negatively, and some of them are not included because they present cross-loadings slightly lower than those of other factors. Although Anjum and Amjad (2021) obtained another four-factor structure from a 72-item version, this was not the official one, but rather they obtained it from their own adaptation of the VIA-240.

Our aim was therefore to test the psychometric properties of the Spanish version of the VIA-72 in a sample of the general population from Spain. Specifically, we aimed to achieve the following: (1) to assess its construct validity by checking the fit of the three-, five-, and six-factor structures; (2) to test its reliability; and (3) to assess its external validity through associations with personality traits.

## 2. Materials and Methods

### 2.1. Sample

The sample consisted of 470 people, comprising 232 women, 152 men, and 84 people who did not indicate their gender, all from Andalusia in the south of Spain. The mean age was 44.79 (SD = 10.48) and the level of education was mainly university-educated (21.77%), while 15.7% had secondary education and 6.6% had primary education. The remaining 44% did not indicate their level of education.

### 2.2. Measures

#### 2.2.1. Spanish Translation of the Values in Action-72

The VIA Institute on Character developed the VIA-72 by selecting the three most consistent items from each of the character strength scales in the VIA Inventory of Strengths (VIA-IS) (Peterson & Seligman, 2004). Many translations into languages other than English have been published. We utilized the Spanish translation. The character strengths included were creativity, curiosity, judgment and open-mindedness, love of learning, perspective, bravery, perseverance, honesty, zest, capacity to love and be loved, kindness, social intelligence, teamwork, fairness, leadership, forgiveness and mercy, modesty and humility, prudence, self-regulation, appreciation of beauty and excellence, gratitude, hope, humor, and religiousness and spirituality. Participants were asked to rate how much each statement represented them on a scale from 1 (very much like me) to 5 (very much unlike me). According to viacharacter.org, the internal consistency reliability of VIA-72 averages at 0.75, with a range from 0.60 to 0.87. In this study, the scores were inverted to facilitate interpretation, meaning that a higher score indicated a greater presence of strength.

#### 2.2.2. Spanish Version of the Five-Factor Inventory (NEO-FFI)

Personality was assessed using the Spanish version of the NEO-FFI. It is a self-report inventory that takes approximately 15 min to complete. It measures the five personality dimensions identified by Costa and McCrae (1992): extraversion, agreeableness, conscientiousness, neuroticism, and openness to experience. The inventory consists of 60 items, with 12 items per domain, rated on a 5-point Likert scale. Respondents indicate the extent to which they agree with each statement about themselves, with response options ranging from 0 (strongly disagree) to 4 (strongly agree). Scores for each personality domain are calculated by summing the responses to the 12 items once the values of the inverse items have been reversed. Thus, a score between 0 and 48 can be obtained for each trait. The higher the score, the greater the presence of the pole that gives the trait its name. The NEO-FFI has demonstrated adequate levels of validity and reliability across diverse populations. The Spanish version also exhibits appropriate indices of reliability and validity, with each of the five domains showing adequate internal consistency (Manga et al., 2004). In this study, the internal consistency of the NEO-FFI was adequate in most of its dimensions, with α = 0.83 for neuroticism, α = 0.79 for extraversion, α = 0.73 for openness to experience, α = 0.77 for conscientiousness, and acceptable for agreeableness of α = 0.66.

### 2.3. Procedure

This study is part of a research project on the relationship between personal strengths and dyadic adjustment in adult couples. To access the sample, directors of family counseling centers and heads of parents’ associations in schools in Cadiz, Andalusia, Spain, were contacted. An advertisement was distributed through them requesting participation in the study. Those who agreed to participate were sent a web link with an information sheet, an informed consent form, and, among others, the two tests described in the previous section. The first author was always available for anyone who requested more information or had questions regarding their participation. This research was conducted in accordance with the Declaration of Helsinki. Data processing adhered to the current regulations, specifically the Spanish Personal Data Protection Act of 1999 and the General Data Protection Regulation (GDPR) of 2016. These regulations always ensure the anonymity and security of the information. This study was approved by the Research Ethics Committee of the University of (anonymized for review) with protocol number (0520-N-22).

### 2.4. Data Analyses

To address the study objectives, we used the factors established in the Spanish version of the VIA-72 (https://www.viacharacter.org/researchers/assessments/via-72, accessed on 7 July 2025). As mentioned in the Measures section, its 72 items correspond to the top three items for each of the 24 character strengths proposed by the theoretical model (Peterson & Seligman, 2004). Some studies have conducted scale-level analyses, using the total scores of the twenty-four character strengths (Moreira et al., 2022), whereas others have opted for item-level analysis (Rodríguez Rueda, 2024). In our study, we chose the latter approach. For example, since items 12, 35, and 62 are theoretically grouped in forgiveness—a strength that, within the three-factor models, is eventually integrated into Caring—we tested in our study whether these three individual items loaded directly onto the Caring factor.

Data were processed using SPSS v29 (IBM SPSS Inc., 2023) and JASP 0.19.2 (JASP Team, 2024). The internal validity of the scale was studied by using confirmatory factor analyses to test the different models using R version 4.4. (R Core Team, 2024), using the lavaan package ‘0.6.17’ for structural equation modeling (SEM). We ran the Unweighted Least Squares (ULS) estimation method applied to the polychoric correlation matrix, which appropriately handled the ordinal structure of the data (Forero et al., 2009). Unlike likelihood-based methods, ULS is a distribution-free estimator that does not assume normality, which enhances the robustness of parameter estimates (Li, 2016). Although Diagonal Weighted Least Squares is also suitable for ordinal data, the high dimensionality of the model with 72 items and the moderate sample size (*N* = 470) led us to run ULS, which tends to be more stable and less prone to convergence issues in complex models, providing consistent parameter estimates (Kline, 2023). Due to the sensitivity to the sample size of χ^2^ of the goodness of fit, we provided the Comparative Fit Index (CFI) and the Tucker–Lewis Index (TLI). Although CFI and TLI values of 0.90 indicated acceptable fit, Hu and Bentler (1999) recommend values of 0.95 to consider a good fit of the data. We also provided the relative indices of fit: the Root Mean Square Error of Approximation (RMSEA) and the Standardized Root Mean Square Residual (SRMR). In this case, values < 0.05 and close to 0.08, respectively, are indicators of good fit (Hu & Bentler, 1999). The internal consistency of all factorial solutions was studied through Cronbach’s alpha (α) and McDonald’s omega (ω). Additionally, we conducted SEM analysis to evidence the construct validity while controlling for multiple comparisons by obtaining standardized beta coefficients (i.e., partial correlations) between each dimension of VIA-72 with the main personality traits obtained with the NEO-FFI.

## 3. Results

### 3.1. Confirmatory Factor Analyses

Table 1 shows the fit data for the three models tested. As can be seen, all three models achieved adequate fit.

**Figure 1:**
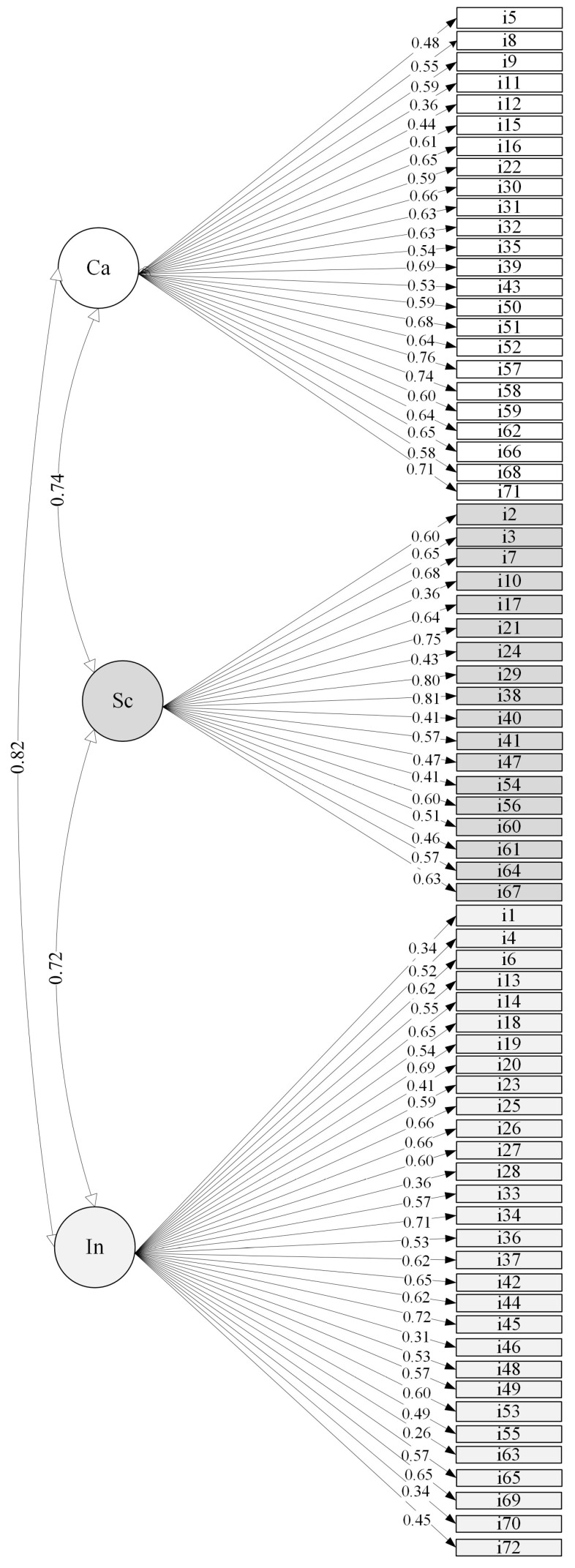
Structure with three correlated factors and standardized factor loadings. Notes: Ca: Caring. Sc: Self-Control. In: Inquisitiveness.

**Figure 2:**
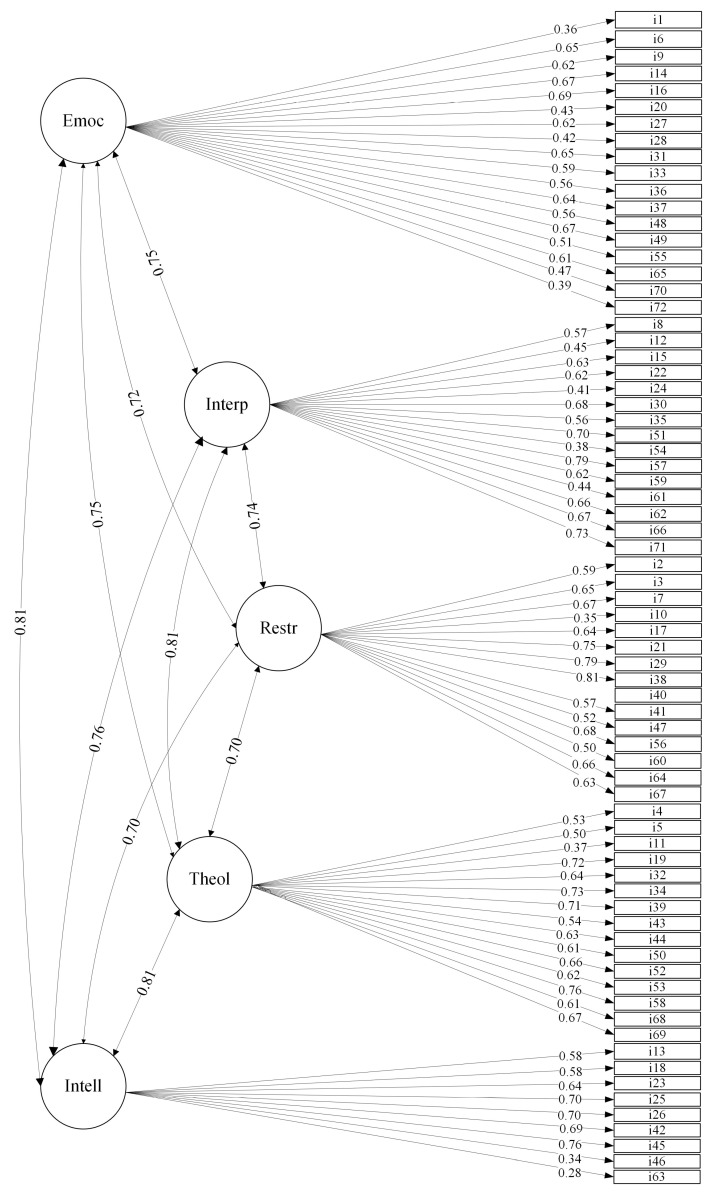
Five-factor correlated solution and standardized factor loadings. Notes: Emoc: emotional strengths. Interp: interpersonal strengths. Restr: restraint strengths. Theol: theological strengths. Intell: intellectual strengths.

**Figure 3:**
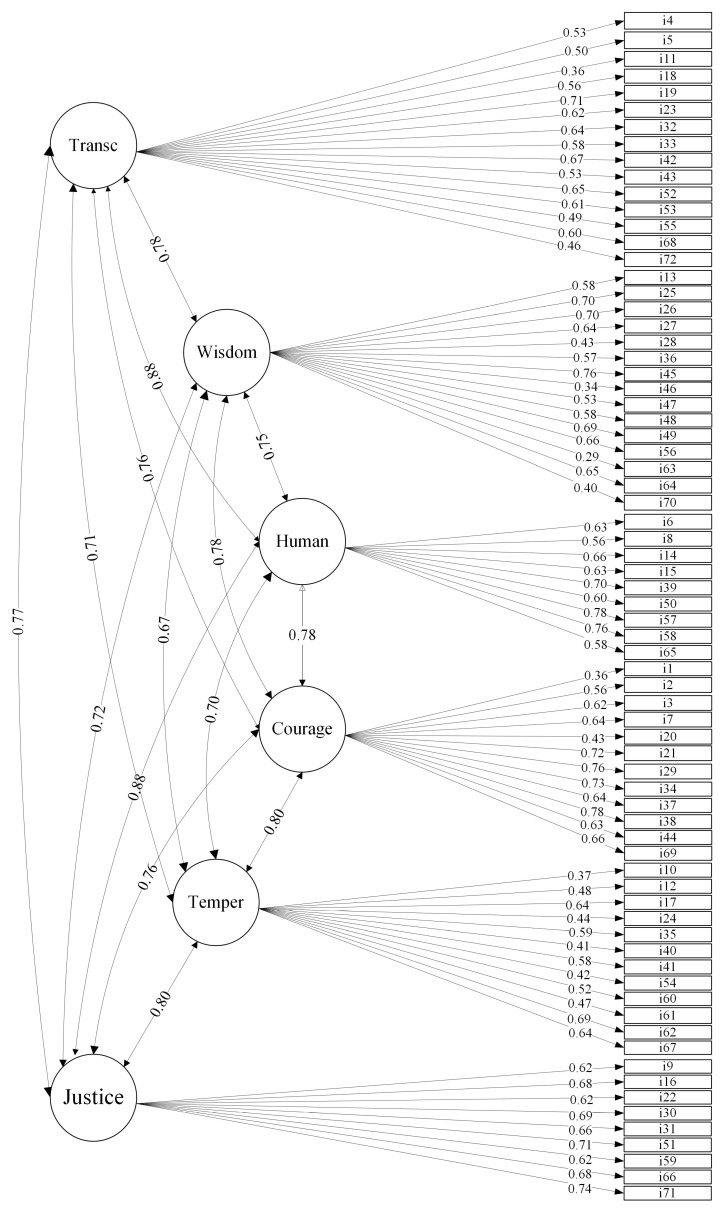
Six-factor correlated solution and standardized factor loadings. Notes: Transc: Transcendence. Human: Humanity. Temper: Temperance.

Figure 1, Figure 2 and Figure 3 represent the three-, five-, and six-factor structures, respectively, using a path diagram. As shown in the figures, the latent factors were named identically to those in the reference studies. The three items that took part of each of the original twenty-four strengths were loaded on the appropriate factors in the three confirmed structures.

In the three-factor structure, Caring groups the 24 items corresponding to kindness, love, teamwork, fairness, leadership, gratitude, spirituality, and forgiveness; Self-Control groups the 18 items corresponding to judgment, perseverance, honesty, self-regulation, prudence, and modesty; and Inquisitiveness groups the 30 items corresponding to curiosity, love of learning, creativity, social intelligence, perspective, bravery, beauty, hope, humor, and zest.

In the five-factor structure, emotional strengths groups the 18 items of creativity, social intelligence, perspective, bravery, humor, and leadership; interpersonal strengths the 15 items of modesty, forgiveness, kindness, teamwork, and fairness; restraint strengths the 15 items of judgment, perseverance, honesty, self-regulation, and prudence; theological strengths the 15 items of gratitude, hope, spirituality, zest, and love; and intelligence strengths the 9 items of curiosity, love of learning, and beauty.

In the six-factor structure, transcendence groups the 15 items of beauty, gratitude, hope, spirituality, and humor; wisdom the 15 items of judgment, curiosity, love of learning, creativity, and perspective; humanity the 9 items of kindness, love, and social intelligence; courage the 12 items of zest, bravery, perseverance, and honesty; temperance the 12 items of self-regulation, prudence, modesty, and forgiveness; and justice to the 9 items of teamwork, fairness, and leadership.

High correlations between the factors were observed in all the factor structures: 0.72–0.82 in the three-factor, 0.70–0.81 in the five-factor, and 0.67–0.88 in the six-factor structure. For the three-factor solution, the items obtained factor loadings between 0.41 and 0.81, except for items i1, i10, i11, i28, i46, i63, and i70, whose factor loadings were lower than 0.40. In the five-factor solution, high factor loadings were also obtained between 0.40 and 0.70, except for items i1, i10, i11, i46, i54, i63, and i70, which did not reach loadings of 0.40. Finally, the six-factor model also had high factor loadings, between 0.40 and 0.78, except for items i1, i10, i11, i46, and i63, whose loadings were below 0.40. Only five items loaded below 0.40 on all three factor structures: i1 (“I have taken frequent stands in the face of strong opposition”, i10 (“Even when candy or cookies are under my nose, I never pig out on them”), i11 (“I practice my religion”), i46 (“I read all the time”), and i63 (“I read a wide variety of books”).

### 3.2. Reliability Analyses

Regarding the reliability analysis, the α and ω coefficients are shown in Table 2. The three models showed good internal consistency in all cases for all dimensions, and excellent for the global scale scores (α_global_ = 0.95, SE = 0.003; ω_global_ = 0.95, SE = 0.003).

### 3.3. Construct Validity

The results of the partial correlations between the dimensions of each model tested and the five personality traits measured with the NEO-FFI are shown in Table 3. Forty-three percent of the correlations were statistically significant. Negative correlations were found between all the strengths that significantly correlated with neuroticism, and positive significant correlations were found with all the other traits. Quantitatively, conscientiousness was the most salient trait, with 86% of its correlations being statistically significant. Fifty percent of the significant correlations had small effect sizes. Forty percent of the significant correlations had medium or close to medium effect sizes, primarily in conscientiousness and extraversion. There were three large or close to large effect sizes (10%), all of which occurred in conscientiousness. Neuroticism and openness to experience had small effect sizes, except for two cases in openness, which were medium-sized.

When examining the three-factor model, conscientiousness is the trait most strongly associated with Self-Control (large effect size). In addition, extraversion is linked to Caring, while openness contributes to understanding Inquisitiveness. In all these latter cases, effect sizes range from small to moderate.

Regarding the five-factor model, conscientiousness is again the trait most clearly associated with restraint strengths (large effect size) and interpersonal strengths. Openness is additionally related to intellectual strengths (moderate to large effect size), and both are complemented by extraversion in explaining emotional strengths. Finally, theological strengths are associated with tendencies towards extraversion and emotional stability.

In the six-factor model, each strength correlates with two or three personality traits, with effect sizes ranging from small to medium. Transcendence reflects an extraverted and open profile; wisdom is linked to a neurotic, open, and conscientious profile; humanity to an extraverted and conscientious profile; courage to a neurotic, extraverted, and highly conscientious profile; temperance to an agreeable and conscientious profile; and justice to an extraverted, open, and conscientious profile.

## 4. Discussion

We aimed to test the psychometric properties of the Spanish version of the VIA-72. Specifically, our aims were as follows: (1) to assess its construct validity by checking the fit of the three-, five-, and six-factor structures; (2) to test its reliability; and (3) to assess its external validity through associations with personality traits.

In terms of model fit, all three solutions demonstrated acceptable to good fit according to standard indices. Table 4 presents the correspondence between the factors, as well as their relationship with the original 24 character strengths. The three-factor model exhibited slightly superior absolute and incremental fit, with lower RMSEA and SRMR values compared to the five- and six-factor solutions. Although the fit indices for the five-factor model were comparable, the six-factor model showed marginally weaker fit across multiple indices, particularly in SRMR.

Reliability analyses further supported the adequacy of all models, with global reliability estimates consistently high. At the factor level, the three-factor model showed strong internal consistency across all dimensions, higher than those reported by Moreira et al. (2022) and comparable to those reported by McGrath et al. (2018). These slightly outperformed the other models, particularly the five-factor model, where the intellectual strengths factor demonstrated relatively lower reliability.

Convergent validity analyses revealed meaningful and theoretically coherent correlations between the character strengths and the Big Five traits across all models. The three-factor structure showed robust associations, overall, with conscientiousness, and particularly between Self-Control and conscientiousness, Caring and extraversion, and Inquisitiveness and openness to experience, all aligning with the prior literature (McGrath, 2015; McGrath et al., 2018). Although not based specifically on the Big Five model, Moreira et al. (2022) also reported similar associations with temperament traits such as novelty seeking and harm avoidance, as well as positive associations with reward dependence and persistence. Additionally, the three character traits—self-directedness, cooperativeness, and self-transcendence—were positively related to the character strength dimensions. Although the five- and six-factor models captured additional nuances, such as theological- and transcendence-related dimensions, the added complexity did not provide a substantially improved explanatory pattern relative to the three-factor structure.

Taken together, these findings suggest that the three-factor model offers the most parsimonious and psychometrically robust representation of the VIA-72 in the current sample. It balances model fit, internal consistency, and meaningful external associations with personality traits, while maintaining theoretical coherence and interpretability. Accordingly, the remainder of the discussion will focus on the three-factor model.

Model fit indices cannot be directly compared, as the reference studies that identified three-factor structures in the VIA-72 (Moreira et al., 2022), VIA-240 (McGrath, 2015), and VIA-120 (McGrath et al., 2018) employed Principal Component Analysis rather than confirmatory factor analysis.

In terms of classification, our results match those of Moreira et al. (2022) in 71% of cases (17 out of 24 strengths). The discrepancies include spirituality loading on Inquisitiveness instead of Caring; perseverance and honesty loading on Caring instead of Self-Control; love of learning and perspective on Self-Control rather than Inquisitiveness; and bravery and appreciation of beauty and excellence on Caring instead of Inquisitiveness. Although McGrath et al. (2018) used the VIA-120, his study remains a key reference due to its unprecedented sample size (N = 1,082,230) and the diversity of its data sources (12 distinct samples). Strengths were classified under a virtue if they loaded on the same factor in at least 10 out of the 12 samples. As in the present study, 17 out of 24 strengths aligned with this criterion: kindness, gratitude, love, teamwork, forgiveness, and leadership loaded on Caring; prudence, perseverance, self-regulation, honesty, and modesty on Self-Control; and curiosity, creativity, zest, bravery, love of learning, and hope on Inquisitiveness. However, four strengths loaded on more than one virtue: beauty on both Caring and Inquisitiveness; fairness on Caring and Self-Control; and both judgment and perspective on Inquisitiveness and Self-Control. In contrast, humor, social intelligence, and spirituality did not load strongly on any factor.

The intercorrelations among the three factors were positive and of a large effect size, consistent with the findings of Moreira et al. (2022), and like those reported by McGrath et al. (2018) where the associations were moderate to large in magnitude.

Despite the strengths of this study, several limitations should be acknowledged. First, the sample was non-probabilistic and included a substantial proportion of participants who did not report key sociodemographic information, limiting the generalizability of the findings. Second, all data were self-reported, which may have introduced response biases such as social desirability. Third, although this study offers a thorough psychometric evaluation of the VIA-72, it does not include comparisons with other abbreviated forms such as the VIA-120 or the VIA-IS-R, which could further contextualize its performance. Moreover, the cross-sectional design precludes any causal inferences regarding the relationships between character strengths and personality traits. This study also did not assess measurement invariance across demographic subgroups, which would be essential to confirm the structural robustness of the model. Lastly, while personality traits were used to support convergent validity, the inclusion of additional external constructs—such as life satisfaction or well-being—would have enhanced the scope of validation evidence.

Future research should seek to address these limitations and further explore the psychometric robustness of VIA-72. Replication studies with larger and more diverse samples, including probabilistic sampling methods, are necessary to enhance generalizability. It would also be valuable to test measurement invariance across demographic groups such as age, gender, and education level to ensure structural consistency. Longitudinal designs could help clarify the causal directionality between character strengths and personality traits or well-being indicators. Moreover, future studies might benefit from incorporating a broader range of external criteria, including measures of psychological well-being, life satisfaction, prosocial behavior, or mental health outcomes. Comparative studies involving different versions of the VIA (e.g., VIA-72, VIA-120, VIA-IS-R) could also help determine which format offers the optimal balance between brevity and psychometric rigor in specific research or applied contexts.

In conclusion, the present study provides empirical support for the Spanish adaptation of the VIA-72, confirming its reliability and validity as a concise measure of character strengths. Among the tested models, the three-factor structure—comprising Caring, Self-Control, and Inquisitiveness—emerged as the most parsimonious and psychometrically robust. These findings align with previous research using both abbreviated and full versions of the VIA inventory, reinforcing the conceptual coherence of this triadic model. The VIA-72 thus offers a practical and theoretically grounded tool for assessing character strengths in Spanish-speaking populations, with promising applications in both research and applied psychological contexts.

## Figures and Tables

**Table 1:** Fit indices obtained from the confirmatory factor analyses.

Model	χ^2^/df	CFI	TLI	RMSEA [90%CI]	SRMR
Three factors	3.03	0.951	0.950	0.066 [0.064–0.068]	0.080
Five factors	3.34	0.951	0.949	0.069 [0.067–0.070]	0.080
Six factors	3.32	0.948	0.946	0.070 [0.069–0.072]	0.082

**Table 2:** Reliability coefficients.

Factor	α (SE)	ω (SE)
**Three-factor model**		
Caring	0.90 (0.008)	0.90 (0.008)
Self-Control	0.87 (0.009)	0.87 (0.009)
Inquisitiveness	0.91 (0.007)	0.91 (0.007)
**Five-factor model**		
Emotional strengths	0.87 (0.010)	0.87 (0.010)
Interpersonal strengths	0.83 (0.012)	0.84 (0.012)
Restraint strengths	0.87 (0.009)	0.87 (0.009)
Theological strengths	0.87 (0.011)	0.87 (0.011)
Intellectual strengths	0.79 (0.017)	0.79 (0.015)
**Six-factor model**		
Transcendence	0.84 (0.011)	0.84 (0.012)
Wisdom	0.86 (0.010)	0.86 (0.010)
Humanity	0.81 (0.014)	0.82 (0.014)
Courage	0.85 (0.011)	0.86 (0.011)
Temperance	0.79 (0.013)	0.79 (0.013)
Justice	0.82 (0.013)	0.83 (0.013)

Notes: α = Cronbach’s α; ω = McDonald’s ω; SE = Standard Error.

**Table 3:** Partial correlations between personality traits (NEO-FFI) and character strengths from the three factor structures (VIA-72) (N = 207).

	N		E		O		A		C	
	r_partial_	SE	r_partial_	SE	r_partial_	SE	r_partial_	SE	r_partial_	SE
Caring	−0.11	0.08	0.21 *	0.07	0.10	0.07	0.06	0.07	0.19 *	0.08
Self-Control	−0.07	0.07	−0.11	0.07	0.04	0.06	−0.01	0.06	0.56 *	0.06
Inquisitiveness	−0.17	0.07	0.22 *	0.06	0.24 *	0.06	−04	0.06	0.29 *	0.07
Emotional	−0.07	0.07	0.25 *	0.07	0.16 *	0.07	−0.05	0.06	0.30 *	0.07
Interpersonal	−0.09	0.08	0.09	0.07	0.13	0.07	0.10	0.07	0.24 *	0.08
Restraint	−0.08	0.07	−0.08	0.07	0.02	0.06	−0.04	0.06	0.58 *	0.06
Theological	−0.22 *	0.07	0.31 *	0.07	0.04	0.07	0.04	0.06	0.15	0.07
Intellectual	−0.13	0.07	0.00	0.07	0.40 *	0.06	−0.04	0.06	0.21 *	0.07
Transcendence	−0.10	0.07	0.30 *	0.07	0.15 *	0.07	0.06	0.07	0.08	0.08
Wisdom	−0.14 *	0.07	0.01	0.07	0.34 *	0.06	−0.12	0.06	0.34 *	0.07
Humanity	−0.12	0.07	0.30 *	0.07	0.12	0.06	−0.06	0.06	0.25 *	0.07
Courage	−0.16 *	0.07	0.16 *	0.06	0.00	0.06	−0.05	0.06	0.49 *	0.06
Temperance	−0.10	0.08	−0.12	0.07	−0.02	0.07	0.14 *	0.07	0.35 *	0.07
Justice	−0.01	0.08	0.10 *	0.08	0.15 *	0.07	0.02	0.07	0.26 *	0.08

Notes: * *p* < 0.05. N: Neuroticism. E: Extraversion. O: Openness to experience. A: Agreeableness. C: Conscientiousness. SE = Standard Error.

**Table 4:** Correspondence of the 24 original strengths with the confirmed factor structures.

Strength	6-Factor	5-Factor	3-Factor
Hope	Transcendence	Theological	Inquisitiveness
Beauty		Intellectual	
Humor		Emotional	
Gratitude		Theological	Caring
Spirituality			
Curiosity	Wisdom	Intellectual	Inquisitiveness
Learning			
Creativity		Emotional	
Perspective			
Judgment		Restraint	Self-Control
Perseverance	Courage	Restraint	Self-Control
Honesty		Restraint	
Zest		Theological	Inquisitiveness
Bravery		Emotional	
Self-regulation	Temperance	Restraint	Self-Control
Prudence			
Modesty		Interpersonal	
Forgiveness			Caring
Kindness	Humanity	Interpersonal	Caring
Love		Theological	
Social IQ		Emotional	Inquisitiveness
Teamwork	Justice	Interpersonal	Caring
Fairness			
Leadership		Emotional	

## Data Availability

The datasets presented in this article are not readily available because they are part of ongoing studies. Requests to access the datasets should be directed to the corresponding author.

## Abbreviations

The following abbreviations are used in this manuscript:

VIA	Values in Action
VIA-IS	Values in Action Inventory of Strengths
CSRF	Character Strengths Rating Form
BSS-12	Brief Strengths Scale
AIVS	Adapted Inventory of Virtues and Strengths
NEO-FFI	NEO Five-Factor Inventory
GDPR	General Data Protection Regulation
ULS	Unweighted Least Squares
CFI	Comparative Fit Index
TLI	Tucker–Lewis Index
RMSEA	Root Mean Square Error of Approximation
SEM	Structural equation modeling
SRMR	Standardized Root Mean Square Residual
